# Silica sol as grouting material: a physio-chemical analysis

**DOI:** 10.1186/s40580-018-0138-1

**Published:** 2018-02-28

**Authors:** Christian Sögaard, Johan Funehag, Zareen Abbas

**Affiliations:** 1 Department of Chemistry and Molecular Biology, University of Gothenburg, Gothenburg, Sweden; 2 Department of Geology and Geo-technique, Chalmers University of Technology, Gothenburg, Sweden

**Keywords:** Silica nanoparticles, Silica gels, Grouting, Gel strength

## Abstract

At present there is a pressing need to find an environmentally friendly grouting material for the construction of tunnels. Silica nanoparticles hold great potential of replacing the organic molecule based grouting materials currently used for this purpose. Chemically, silica nanoparticles are similar to natural silicates which are essential components of rocks and soil. Moreover, suspensions of silica nanoparticles of different sizes and desired reactivity are commercially available. However, the use of silica nanoparticles as grouting material is at an early stage of its technological development. There are some critical parameters such as long term stability and functionality of grouted silica that need to be investigated in detail before silica nanoparticles can be considered as a reliable grouting material. In this review article we present the state of the art regarding the chemical properties of silica nanoparticles commercially available, as well as experience gained from the use of silica as grouting material. We give a detailed description of the mechanisms underlying the gelling of silica by different salt solutions such as NaCl and KCl and how factors such as particle size, pH, and temperature affect the gelling and gel strength development. Our focus in this review is on linking the chemical properties of silica nanoparticles to the mechanical properties to better understand their functionality and stability as grouting material. Along the way we point out areas which need further research.

## Introduction

Silica sols have increasingly been used in diverse applications in several different areas, one of which is the field of grouting. Grouting of strata can have different purposes such as stabilization of loose sands, preventing leakages etc. One area that has been explored for some years is the permeation grouting (or grouting of rock) using silica sol [1]. In Scandinavia the permeation-grouting is a primary method used to prevent the leakage of water into tunnels and hard rocks. It requires a grout to be applied into the leaking cracks in order to physically block the water from entering the tunnel. For the grout to be effective it must thus enter cracks of varying size, which puts demands on the rheological properties of the grout. The grout must also once applied, last for a considerable time and thus be stable in the conditions experienced in the rock, primarily against water of different compositions containing various types of ions and pH levels.

Cement based grouts are by far the most common materials used for grouting of rock. However, cement is limited by its particle size to enter very fine fractures and underperformes for strict demands on ingress to tunnels. Better penetration can be achieved by using the so called chemical grouts. Generally materials characterized as chemical grouts are polyurethane, epoxy resins, acrylamide, methacrylate and acrylate [2, 3]. Moreover, water glass and a number of composite materials such as polyurethane/water glass [4], epoxy resin/sand [5], acrylamide/sand [6], have also been used as grouting materials. Although, there is lot of experience in controlling the viscosity, achieving the desired penetration length, desired gel time and good sealing of the small cracks or pores by using organic molecules based grouts, the big issue is their toxicity, As shown by Svedrup et al. there was considerable leaching from the acrylamide based grout used in a tunnel in Norway [7]. In Sweden in the swells of environmental catastrophe involving acrylamide based grouting material in Hallandsås tunnel environmental compatibility of grouting material is highly prioritized. Silica nanoparticles are considered to be non-toxic, mainly due to their instability in groundwater, which is of utmost importance for sustainable development [8, 9]. Therefore, in rock grouting silica nanoparticle based grouts can work as alternative to the organic molecules based grouts since high penetrability can be achieved due to the small particle size.

Industrial scale methods have been developed to produce silica particles of specific size, reactivity and surface area. Today, several silica sols are commercially available and two of the most commonly used are the Ludox^®^ series produced by Grace and the Levasil series produced by AkzoNobel. The diameter of silica particles commonly ranges from 5 to 22 nm although some commercial sols with an average size up to 100 nm are also available [10]. Commercially available silica sols are usually stabilized by sodium ions (Na^+^) at a pH around 10. The properties of the silica sols are of importance to achieve the desired viscosity, which is an important parameter to achieve good grouting. For example silica particles can infiltrate cavities and cracks with a hydraulic aperture of 14 µm, which can be compared with the 0.05–0.1 mm crack-penetration of, the most common grouting material, cement [11].

Silica sols form a gel in the presence of an accelerator [12]. A commonly used accelerator for inducing gelling in silica sols is NaCl salt solution. The induction of gelling is controllable and the gelling can be set to occur instantly or up to several hours which is often required for applications under specific conditions. Other salts such as KCl and CaCl_2_ have also been used as accelerators but not to the same extent as NaCl.

Field experience in using silica sols as grouting material in Scandinavia is limited to the use of NaCl or CaCl_2_ as accelerators [13, 14]. In the case of NaCl, a 10% solution of salt is used to induce gelling whereas gelling occurs at much lower concentration of CaCl_2_. Moreover the gelling using CaCl_2_ is fast and requires slower pouring in the mix. Some practical routines have been worked out from field experiences. For example, a typical mixing ratio by volume is 5:1 which means five parts of silica sol and one part of sodium chloride solution. Using the Levasil CB22, found in Table 1, this results in gelling in 30 min. The grouting continues until at least half the gel time (15 min), and the grouting procedure can be stopped.

**Table 1:** Shows a collection of particle types and their properties as industrially manufactured by Grace or Akzo Nobel

Manufacturer	AkzoNobel	AkzoNobel	AkzoNobel	AkzoNobel	Grace	Grace	Grace	Grace
Type	Levasil CB17	Levasil CB22	Levasil CB25	Levasil CB75	Ludox^®^ HS	Ludox^®^ TM	Ludox^®^ SM	Ludox^®^ TMA
Particle size (nm)	16	12	11	4	12	22	7	22
Stabilizer	Na^+^	Na^+^	Na^+^	Na^+^	Na^+^	Na^+^	Na^+^	–
Concentration SiO_2_ (wt%)	40	40	30	15	30 to 40	40 to 50	30	34
Surface area (m^2^/g)	170	220	250	750	210 to 220	140	320 to 400	140
pH	9.4	9.8	10	10	9.2 to 9.9	9	9.7 to 10.3	4 to 7
Density (g/cm^3^)	1.3	1.3	1.2	1.1	1.3	1.3 to 1.4	1.22	1.23
Viscosity (cPs)	6	15	5	13	< 45	< 10 to < 50	5.5 to 5.7	< 15
Zeta potential (mV)	–	–	–	–	− 30	− 40	–	–

Lacking information is marked with (–). The particle size has been calculated using industrial standard where 2730/surface area = size (nm)

As pointed out by [2], the desirable properties of a good grouting material are controllable gel time, low initial viscosity and durability. The durability of grouted silica depends on both mechanical and chemical interactions. The design methodology used for grouting is also an important factor. As pointed out by [11, 12] knowledge about the fracture apertures to be sealed is required and governs the applied pressure in order to achieve maximum penetration length in the rock cracks. Temperature has a considerable effect. For example gelling time is doubled when temperature is halved [15]. This means that in order to have proper execution of a demanding grouting (such as a post grouting where the hydraulic gradient can be large) the design needs to incorporate the temperature. To achieve efficient grouting the grouted silica sol volume must be optimized by taking account of the applied pressure and gel time. The longer the gel time, and the higher the pressure, the longer is the penetration length and hence the grouted volume is increased.

The experience of grouting with silica sol is that it seals the fractures and lowers the ingress of water to facilities under ground, such as tunnels. However, for some projects with focus on research, some boreholes have not been sealed. The gel in the borehole has been flushed out or just become a mush. Two possible reasons for this are put forward. The hydraulic gradient can have been so high that it creates a “backflow” in the grouted fracture, resulting in a diluted gel. Or the chemical properties of the water present in the rock have been affecting the gel. The pH of the water could have been affected by previous cement grouting or the ion composition of the ground water, could have affected the gelling.

In this review paper we shall address the question of long term stability and functionality of the grouted silica. We shall summarize and analyse the state of the knowledge of major factors affecting the gelling process such as the chemistry of accelerator interaction with the silica nanoparticle surface, ionic composition and pH of the surrounding environment, particle size and surface area. We shall try to correlate these factors with the knowledge about the workability of grouted silica gels wherever it is possible.

## The silica sol (suspension of silica nanoparticles)

The chemical formula of silica is written as SiO_2_ which is correct with regard to the ratio of atoms. However, this does not show the true structure of the atoms in the particle where every Si-atom is tetrahedrally coordinated with O-atoms as a SiO_4_ unit. The Si-atoms are connected by siloxane bonds (Si–O–Si) which form an amorphous network [16].

### Size, surface area and stabilization

The production of silica nanoparticles and silica sols can be considered to be more or less a mature science. Several companies have mastered the art of producing silica sols on an industrial scale, with Grace (formerly DuPont) and AkzoNobel being two of the largest. The particles they produce varies very little from a chemical point of view, the bulk of the particle is always SiO_2_. However the stabilizers, particle size, particle concentration, and surface composition may vary as shown in Table 1.

The surface area of nanoparticles varies with particle size, decreasing with an increased particle size. Nanoparticles have a very large surface area and the number of atoms located at the surface is very large in comparison to bulk-sized materials. The surface of the nanoparticle thus makes up a significant ratio of the particle. The increased reactivity of nanoparticles compared with bulk material is due to the presence of large number of under coordinated oxygen atoms on the surface and thus the surface energy of a nanoparticle is very high. When a nanoparticle interacts with other molecules in solution it tries to minimize the surface energy. This is the reason why silica nanoparticles are strongly hydrophilic. The tendency of nanoparticles to aggregate in solution is also a manifestation of the particles reducing their surface area and thus their high energy state.

The stabilization of the silica particles is mainly dependent on the composition of the particle surface. Common stabilizers are either Na_2_O or substitution of (Si(OH)_4_) with (Al(OH)_4_) during particle production. The aim of the stabilization is to reduce the increase in viscosity, which is otherwise experienced in concentrated silica sols due to the strong hydrophilicity. Silica sols usually have a pH of 10 and at this pH the particles are negatively charged. The stability of particles is achieved by controlling the amount of Na^+^ as counter ions which are produced by the dissociation of Na_2_O. An optimal stability is achieved by perfectly balancing the negative charge on the surface, therefore the amount of Na^+^ ions can be varied depending on the number of charged groups present on the surface [16]. Substituted silica nanoparticles i.e. particles where some silica atoms have been substituted by aluminium atoms are also commercially available. The substitution of aluminium at the surface has the effect of adding negative charge to the surface, which is pH independent and renders the particles stable at low pH i.e., < 8 [10]. On the other hand silica sols using Na_2_O as stabilizers are kept at a pH around 10 where the surface charge is high and the electrostatic repulsions are large.

**Fig. 1:**
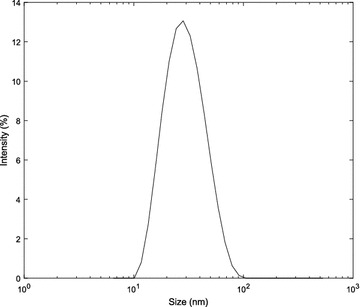
Example of particle size distribution for a Ludox^®^ TM-40 silica sol from DLS measurement. Note that the x-axis is logarithmic. The distribution is a classical lognormal with particle size from 10 to 100 nm

The size of silica nanoparticles can vary from the smallest with an average diameter of 4 nm to the largest close to the size of bulk materials which is approximately 1 µm. Colloidal particles are limited to a maximum size of 100 nm since sedimentation sets in at larger sizes. The particle size distribution can vary among different types of commercial sols but also within the same type of sols. In Fig. 1 the particle size distribution for a commercially available Ludox^®^ TM-40 sol can be seen. The particle size distribution is a factor that is easily overlooked given that the average particle size, often provided by distributors, says nothing about the size distribution. The distribution of particles is difficult to control and often the specific surface area is used instead. The specific surface area is easier to control and the average particle size is calculated from this using an industrial standard formula; 2730/surface area = size (nm).

### Surface charge and zeta potential of silica

The surface of silica nanoparticles is covered by silanol (SiOH) and siloxane (Si_2_O) groups [16]. The siloxane groups are most prevalent in fumed silica and these groups are often considered to be inert towards deprotonation. The silanol groups are very common at the surface of amorphous silica and their acidity leads to the surface of silica nanoparticles being more or less charged depending on the pH of the surrounding environment; according to the following reactions:

1 $$SiOH \mathop \leftrightarrow \limits^{K1} SiO^{ - } + H^{ + }$$

2 $$SiOH + H^{ + } \mathop \leftrightarrow \limits^{K2} SiOH_{2}^{ + }$$

where reaction (1) is the prevalent reaction at pH ranges from 2 to 12 [17–20]. It has been shown that the surface of silica nanoparticles contains at least two types of silanol groups with pKa values of around 4.5–5.5 and 8.5–9.9 [21, 22]. The first type constitute around 15–19% while the second type constitute around 81–85% of the silanol groups. While the pKa values suggested above were experimentally determined and simulations with ab initio molecular dynamics have shown that there are two types of silanol groups present on the silica surface [23]. Isolated silanol groups having a large distance to the closest neighbouring groups and concave geminal or vicinal groups with only a few Ångström away from neighbouring silanol groups. The geminal and vicinal groups can thus form hydrogen bonds between each other. The simulations results show that isolated and convex germinal silanol groups have high pKa values of around 10 while silanol groups that interact directly or through water bridging with other silanol groups have a pKa of around 2–3. The explanation given is that the negative charge created when a silanol group dissociates is stabilized by the presence of other neutral silanol groups and dissociation is thus facilitated. Isolated groups lack this stabilization and are thus less inclined to dissociate leading to a higher pKa.

Reaction (2) is only active at pH below 0 due to the very low logK_2_ (logK_2_ = − 1.8 to – 1.0). The silanol groups are able to form siloxane bond in accordance with condensation reaction shown in reaction (3) and these bonds are partly responsible for strength development in gels [24]. Carroll et al. [25] have shown by using the ^29^Si NMR spectroscopy that siloxane bonds on silica surface increases with increasing the pH. It was suggested that reaction (3) is the net reaction and that the dissociation of a silanol group is required for the reaction to proceed according to; $a.\,SiOH \to SiO^{ - } + H^{ + } ;\,b.\,SiO^{ - } + SiOH \to SiOSi + OH^{ - }$

3 $$SiOH + SiOH \to Si{-}O{-}Si + H_{2} O$$

**Fig. 2:**
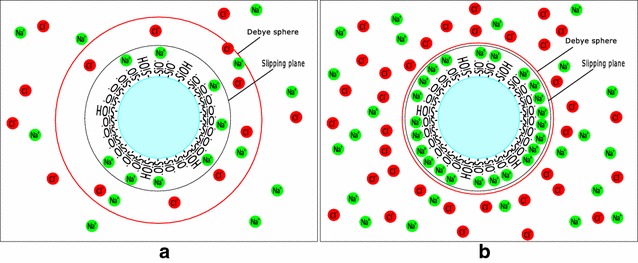
Conceptual picture showing a single silica nanoparticle with a mostly dissociated surface surrounded by an alkali water solution. The sodium ions inside the slipping plane are completely locked in place and make up the stern layer. **a** Represents a particle dissolved in low concentrated NaCl solution and therefore have several unshielded SiO^−^ surface groups. This particle retains most of its electrostatic repulsion forces which is shown by a large Debye sphere and is therefore either stable or will aggregate at a significantly slower rate than the particle shown in **b**. **b** Represents a particle that is dissolved in a highly concentrated NaCl aqueous solution and is completely covered by sodium ions which leads to a greatly reduced Debye sphere. The b particle can be considered to be unstable i.e. it would aggregate with other particles

Dissociation of the silanol groups at pH levels above the point of zero charge (pzc) is the main cause of the negative charge of silica surfaces. The pzc for silica is considered to be between pH 2–4, depending on the silica type [26]. The negative charge will attract cations which form a layer close to the surface of the silica, called the stern layer as described by the stern model [27, 28]. The ions in the stern layer have limited diffusivity due to electrostatic interactions with the silica surface. The area outside the stern layer is known as the diffuse layer and the ion distribution is described by the Gouy–Chapman theory, which is based on Poisson–Boltzman statistics [29]. In the diffuse layer ions can move freely [30]. The interface between the stern layer and the diffuse layer also known as the slipping plane, see Fig. 2, caries a potential known as the zeta potential, which can be measured using electrophoretic mobility methods. The zeta potential is generally used as an indicator of the stability of colloidal solutions. Usually high negative or positive values i.e. > ± 20 mV of the zeta potential are related to a more stable colloid suspension due to strong electrostatic repulsion between the particles. However, it has to be mentioned that a balance has to be maintained because negative or positive zeta potential greatly increases the viscosity of concentrated silica sols [31]. Usually zeta potential of commercial silica sols with a 30–40% particle concentration is kept at a value around − 30 to − 40 mV with the help of the stabilizers as was discussed in “Size, surface area and stabilization” section. The thickness of the double layer around a charged surface including Stern and diffuse layer is described by the screening parameter so called Debye–Hückel kappa (κ) and the inverse of kappa is the screening length usually called Debye screening length which decides the size of the Debye sphere, see Fig. 2. At increasing salt concentration the packing of counter-ions near the surface increases, resulting in the double layer becoming more compact and this result in a decrease of the Debye sphere. The same is true for the zeta potential, which decrease or increase in proportion to the screening and surface charge of the silica.

**Fig. 3:**
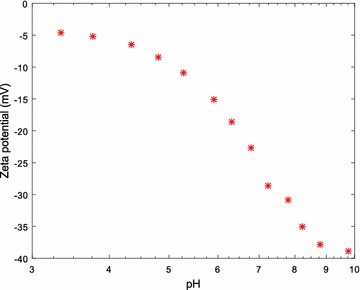
Zeta potential change with pH for Ludox^®^ TM-40 silica sol. The graph shows a clear trend where zeta potential decreases with increase in pH

Measurement of zeta potential is one way of quantifying the electrostatic repulsion forces and can also be used to measure the efficiency of cations to screen a negatively charged surface. It is well known that the zeta potential increase when the concentration of cations increases. However, the concentration at which the zeta potential approaches zero value is highly salt specific. Generally, the more an ion is able to adsorb in the stern layer, the more it affects the zeta potential. As shown by Franks [32] that monovalent cations follow the so called Hofmeister series in their ability to affect the zeta potential. Li^+^ ions adsorb weakly at silica/solution interface and show the most negative zeta potential while Cs^+^ adsorbs strongly and show the most positive zeta potential, at equal ion concentration and at a pH interval of 2–10. Furthermore, ion concentrations was shown to have no effect on the relative position of the ions in the Hofmeister series, with one exception being simultaneous high ion concentration (0.4 M) and low pH (< 4). The Hofmeister series will be further discussed later in Sect. 3.1 of this review. As can be seen in Fig. 3, pH also affects the zeta potential since change in pH affects the surface charge of the silica particle according to reactions (1) and (2).

The zeta potential could provide information about which accelerator to be used in order to achieve the specific aggregation behaviour, because it is one of the few measurable parameters describing the charge at the particle/solution interface. Since it has been shown by Kobayashi et al. [33] and more recently by Ovanesyan et al. [34] that the zeta potential is dependent on the particle size; it could also be used to determine what particle size is optimal in combination with a certain accelerator to achieve aggregation.

From the discussion given above we can summarise that zeta potential, since it is easily measureable, can be used as a simple mean to predict the stability of silica nanoparticles. It can also be used to find an optimal salt concentration to induce gelling by hinting at the kinetics of gelling. Such information is highly desirable in optimizing the grouting strategies for different types of silica and accelerators used for gelling.

### Interaction of ions with silica surfaces

Salts dissolved in water contain ions that once dissociated can be divided into two groups, namely structure breaker or structure maker ions. Structure breaker or maker depends on the ions ability to structure water molecules in close proximity to the ion. In the periodic table group of common accelerators, the alkali metals, lithium and sodium ions are considered to be of structure maker character while potassium, rubidium, and cesium are considered structure breakers. Surfaces such as those of silica nanoparticles can also be divided into structure maker or breaker [21].

The ability of ions to adsorb at a surface can be understood by examining the state of hydration. The structure maker ions, lithium and sodium are characterised by the presence of a hydration layer surrounding the ions. The water molecules around cation are oriented by oxygen atoms pointing towards the ion. The water beyond this hydration layer has its continuous structure.

**Fig. 4:**
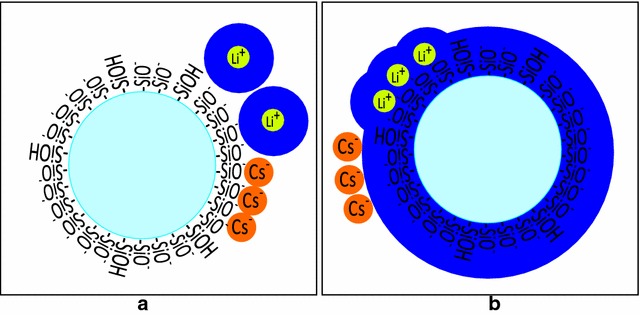
**a** The Hofmeister theory where silica is a structure breaker surface and do not have a ordered hydration layer which allows structure breaker ions to adsorb closer than structure maker ions. **b** The theory suggested by [37] where the silica surface is a structure maker surface and thus has a ordered hydration layer which allows structure maker ions to adsorb more closely to the surface

The number of water molecules in the hydration layer is dependent on ion type [35, 36]. The crystallographic radius of lithium ion is 0.069 nm and is the smallest of the alkali ions. This means that the positive charge of lithium is concentrated on a small area. In water it polarizes water molecules strongly and accumulates several water molecules around it [35, 37, 38]. The Dielectric spectroscopy and Monte Carlo Simulations have shown that Li ion has 8–10 water molecules whereas cesium has no strongly bound water molecule [39, 40]. Therefore, when the hydration shell is included the radius of the lithium ion exceeds that of the otherwise much larger cesium ion [37]. This has an impact on the ability of the lithium ion to approach a silica surface. To be able to predict the lithium ions ability to adsorb to the silica surface, knowledge about the structure of the water around the silica surface is also needed. In literature it has been claimed that the silica surface is a structure breaker surface and that no significant hydration layer exists around it; others have claimed that this is not the case [41]. Colic et al. [37] have shown that silica gels with the highest viscosity are produced by the use of lithium as accelerator. This is not expected since lithium has the highest critical coagulation concentration (ccc, concentration at which particle aggregation occur) of all the alkali metal ions. The experiments were conducted at very high ion concentrations at which all the ions tested totally covered the silica surface. At such conditions the ability of the different ions to adsorb to the surface, and thus the ccc of the ions, matters little. The explanation given for the higher viscosity of lithium accelerated gels is that due to the presence of a hydration layer at the silica surface the lithium ions are able to adsorb more closely to the surface, lowering the distance between particles and thus creating a stronger bond between particles. Again, this is completely opposite the traditional explanation for the Hofmeister series in which the silica surface is regarded as a structure breaker surface lacking of a hydration layer, see Fig. 4. Since in [37] the experiments were conducted at high ion concentrations and in such high salt concentrations even strongly hydrated ions lose their hydration due to the fact that oppositely charged ions come close enough to form ion pairs. Therefore, according to the authors opinion Colic’s data cannot be used to distinguish if lithium is structure breaker or maker ion. The presence of a hydration layer at the silica surface supported by theory [37] seems to have been confirmed by experiments [42].

The sodium ion is the second smallest of the alkaline ions. Like the lithium ion it has a hydration layer of ordered water molecules. According to dielectric spectroscopy and MC simulations it has 4–5 water molecules [39, 40]. In the Hofmeister series it has the second highest ccc, which is logical in the perspective that it has the second highest amount of water molecules surrounding it. Sodium, together with potassium, is due to its availability the most common of accelerators used.

The structure breaker ions usually refer to the monovalent potassium, rubidium, and cesium ions. These ions lack the hydration layer i.e., no strongly bound water molecules around these ions were found by dielectric spectroscopy and MC simulations [39, 40]. The structure breaker ions are known to adsorb to silica surface in accordance with the Hofmeister series. Their ability to adsorb to the silica surface to a higher extent than structure maker ions at low concentrations is due to their ability to approach the silica surface much closer since they lack a hydration layer. However, recent studies showed that the Hofmeister series inverted for monovalent cations. It is interesting to note that in these studies large silica particles approximately 2.5 μm or larger were used and the inversion was observed as pH was increased above pH 7–10 [43–45]. Such an inversion can be due to differences in the properties of large crystals such as porosity and density of surface sites compared to the nanoparticles. The particle curvature has profound effect on the accumulation of counter ions near the surface of nanoparticle and consequently increases the surface charge density of nanoparticles compared to the large particles. This is an interesting matter which needs to be investigated in detail.

Franks [32] found that the highest yield stress of silica gels was achieved by using structure breaker ions as an accelerator at pH 6–10. The yield stress follows the Li < Na < K < Rb < Cs at pH 6–10 and 0.4 M salt concentration but below pH 5 it inverts so that lithium leads to the highest yield stress. It was noted that if salt concentration increase the pH at which inversion occurs also increase.

Another phenomenon linked to structure breaker monovalent ions as well as structure maker divalent ions (Ca^2+^) is the charge inversion of the silica surface [32, 46, 47]. At a certain accelerator concentration the silica surface will be covered by ions to such an extent that the surface has no charge. That is, the screening is so effective as to completely hide the charge of the surface. At this point the zeta potential will be equal to zero and the Debye sphere will not be present, meaning that no electrostatic repulsion exists between the particles. If the accelerator concentration is high enough, both potassium and cesium have been observed to continue adsorbing to the surface even after complete screening is achieved. This leads to a overcharging of the surface with a positive surface charge as a result. The electrostatic repulsion forces not present or severely reduced by the presence of the accelerator can thus re-emerge due to the higher numbers of ions at the silica surface [48, 49]. The forces between particles in presence of different ions is treated in detail in a review by Trefalt et al. [50].

## Gelling of silica sols

The gradual build-up of the gel is achieved through the aggregation of silica nanoparticles into larger and larger aggregates [16, 38]. The aggregation is initiated by the addition of an accelerator to the silica sol as described in the preceding section and the mechanism of destabilization and aggregate formation can partly be described by the Derjaguin–Landau–Verwey–Overbeek (DLVO) theory. Eventually the aggregates have grown to such a size that a continuous network is formed and at this point the silica can be considered a gel. Whether the point at which the continuous network is formed is the same as the gel point can at present not be confirmed, but the authors would argue that it is not an unreasonable thought.

The DLVO theory has long been the prevalent theory used in describing the stability of colloidal systems. It describes the stability of particle suspensions through a balance between repulsive electrostatic forces and attractive van der Waals forces. If repulsive forces dominate, the suspension is stable, and if attractive forces dominate, the suspension becomes destabilized. The repulsive electrostatic forces are described by Poission–Boltzman theory whereas attractive forces are described by the Hamaker theory [31]. A large value of the Hamaker constant lead to strong attractive forces between two materials and vice versa. Silica nanoparticles have a relatively low Hamaker constant ($6.5 \times 10^{ - 20} \,{\text{J}}$) compared to other oxides ($TiO_{2}{:}15.3 \times 10^{ - 20} \,{\text{J}}, \;\alpha - Al_{2} O_{3}{:} 15.2 \times 10^{ - 20} \,{\text{J}}$), which mean that the van der Waals forces are weak between the silica particles [51]. The electrostatic repulsion forces are described by the surface potential of charged particles. Since surface potential hard to measure, zeta potential is often considered as surface potential in DLVO calculations. The general prediction of the DLVO theory is that when surfaces are sufficiently charged i.e., at pH values far from the pzc the electrostatic repulsive forces stabilize the charged particles. However, when particle surfaces are neutral van der Waals attractive forces dominate and induce aggregation.

For silica sols the DLVO theory does to a large extent describe the stability of nanoparticles at pH ranges from 8 and above. However, the theory has received much scrutiny due to its inability to explain the behaviour of the particles at lower pH levels, especially at pH levels close to the pzc where silica particles are stable despite the absence of electrostatic repulsion. This has led to the introduction of non DLVO interactions, which include theories regarding the importance of the structure of the water surrounding the particles and the so called gel layer thought to be present at low pH. In the literature some other limitations of the DLVO theory have been pointed out. Kobayashi et al. [33], have shown that for large silica nanoparticles (80 nm) the DLVO theory successfully predict the aggregation behaviour of the particles but for small to medium silica nanoparticles (20–40 nm) this is not true. Small particles were stable at low pH (< 6) which is not in accordance with DLVO theory where the stability should decrease as a result of decreased surface charge. It was suggested that additional repulsive forces, not described by DLVO theory must be present in order to describe the stability of the smaller nanoparticles at low pH levels. These additional forces are assumed to be due to a gel layer of poly(silicilic acid) extending a few nanometres from the silica surface, presenting a mechanical obstacle to particle aggregation [33, 38]. This gel layer mostly affects smaller nanoparticles due to the larger amount of surface area compared to larger nanoparticles. The gel layer is only present at low pH due to the dissolution of poly(silicilic acid) at pH values above 6. These observations prove that it is difficult to describe the stability of silica nanoparticles purely through theoretical models such as the DLVO theory but that these models need to be complemented with experiments that yield information about the behaviour of particles. A review that describes these non DLVO interactions of colloidal systems in detail has been published by Grasso et al. [52].

**Fig. 5:**
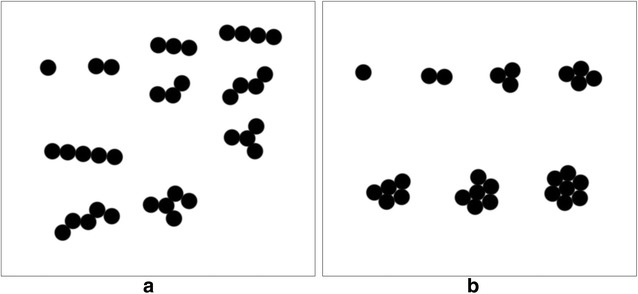
**a** Examples of aggregates formed in DLCA aggregation while **b** shows examples of aggregates formed in RLCA. Note the difference in aggregate density between **a** and **b**, where **a** forms less dense aggregates, arising from the different aggregation mechanisms

An often discussed phenomenon is the slow versus fast aggregation mechanism [53–56]. Fast aggregation, also known as diffusion-limited cluster aggregation (DLCA), occurs at high ion concentration and is more pronounced for the structure breaker ions. In DLCA every particle collision results in aggregation and the rate limiting step is the number of particle collisions which are coupled to particle diffusion. In addition to the high accelerator concentration increased valency of the accelerator can also lead to fast aggregation. In DLCA aggregates can in principle take on any form and structure since every single particle collision leads to aggregate formation, see Fig. 5a.

Slow aggregation, also known as reaction-limited cluster aggregation (RLCA), occurs at low ion concentration and is more pronounced with structure maker ions as accelerators; in that these ions have a larger concentration span at which slow aggregation occurs. In RLCA every collision between particles does not lead to aggregation which results in the interaction between particles being the aggregation rate limiting step. This means that the point at which a particle and particle/aggregate collide is of utmost importance to RLCA. For example a particle will only stick to other particles/aggregates if the interaction between the two particles is sufficient, see Fig. 5b. Often these interactions decrease with the surface area of the particles.

In DLCA and RLCA the particle size may affect the aggregation and gelling behaviour since the amount of surface area is directly coupled to the particle size, although surface roughness may also affect the surface area. For example, as discussed above larger particles have shown to behave more in accordance with DLVO theory than smaller particles [33]. Furthermore the ccc have been shown to vary with particle size [57]. The increase in surface area of smaller particles lead to more counter ions being needed to achieve screening in order to start aggregation. However, once aggregation has started smaller particles will aggregate faster due to their increased diffusion speed [58].

### Effects of ion type on the gelling of silica

As discussed in preceding paragraphs, salts, known as accelerators, can be used to induce the aggregation of silica sols by destabilising the silica nanoparticles [38, 53, 59]. Aggregation of the particles eventually leads to the formation of a particle network at the point of gelation (PoG). It has been shown in literature that the gelling of silica follows the previously mentioned Hofmeister series [29, 60]. The concept of Hofmeister series has been known ever since it was established by Hofmeister roughly one century ago [61]. At the time Hofmeister observed the salting out (precipitation) of protein in egg white as an effect of the addition of ions. Therefore the first Hofmeister series was for the destabilization of egg white proteins when anions were introduced. Since its formulation the concept of Hofmeister series has undergone constant refinement and development. Today Hofmeister series exists for several surfaces and includes series of cations as well as anions [62–64].

Since silica sols are negatively charged the Hofmeister series for these surfaces is based on cations. For monovalent cations it corresponds to Li^+^< Na^+^< K^+^< Rb^+^< Cs^+^ where Li^+^ is the least effective and Cs^+^ is the most effective destabiliser (accelerator). It is important to note that the Hofmeister series is based on the concentration of ions needed to reach the ccc; it says nothing about the final properties of the resultant silica gel. However, it does offer explanations to what governs the gelling of silica sols and points towards the importance of cation adsorption to the silica surface. It has also been shown that divalent ions, such as Ca^2+^, have a lower ccc than monovalent ions [41]. This is due to their higher charge which increases their attraction towards the silica surface. In a recent study [65] it has been claimed that varying the counter ions changes the kinetics of gel formation of silica particles but not the structure of final gel. Most interestingly the author’s claim that gels produced by Na and K as accelerators develop to the same structure. Therefore the differences between gels generated by different salts are transient in time.

For aluminium surfaces the Hofmeister series has been observed to be the opposite of silica [32, 64]. This is of special interest since some silica particles are stabilized using aluminium substitution. The effect that this has on the accelerator efficiency on the silica particle aggregation is unknown to the authors. One speculation is that the Hofmeister series of silica nanoparticles approach that of aluminium as more silica is substituted with aluminium.

### Effect of pH on gelling

The surface of silica nanoparticles are affected by the pH when dissolved in aqueous solution, as described previously. As pH is increased, silanol groups dissociate and the surface becomes more negatively charged. Silica nanoparticles experience a stability minimum at pH 6–7 and from this point increase in pH lead to increased stability leading to either longer gel times or a need to increase accelerator concentration in order to achieve gelling [66]. Silica nanoparticles experience a stability maximum at the pzc. This is thought to be due to either, as previously discussed, the presence of a hydration layer or a silicic acid layer at the surface, which prevents the particles from approaching each other. It would be logical to assume that since the silica surface is uncharged at the pzc, accelerators would have no effect on the behaviour of the particles at the pzc. However, it has been shown [37] that although no change occurs over time at the pzc, accelerators have an effect on the viscosity of the particle solution. This is not in accordance with DLVO theory and thus indicates the presence of non-DLVO interactions. The explanation given is that the ions snatch water molecules from the silica surface which leads to a breakdown of the hydration layer that would otherwise prevent aggregation. Lithium shows the highest viscosity due to its large affinity for water molecules while cesium shows the lowest viscosity due to its low affinity for water molecules. This explanation assumes the presence of a hydration layer on the silica surface and thereby assumes silica to be a structure maker surface.

At the other end of the pH spectrum, at high pH values above 11 some intriguing accelerator effects on the gelling of silica nanoparticles have been observed. Above pH 11 it has been observed that the structure breaker ions, potassium, rubidium, and cesium do not induce gelling, while the structure maker ions, lithium, and sodium do [24, 67]. The structure maker and structure breaker ions are antagonistic in their behaviour and the introduction of structure maker ions induces gelling while introduction of structure breaker ions leads to peptization (breakup of aggregates) of the gel. The process is reversible in that the ratio between structure maker ion concentration versus structure breaker ion concentration seems to govern which of the two processes dominate. However, it remains to be answered whether this behaviour can be observed with gels that have been allowed to age and which should have formed covalent siloxane bonds between the particles incorporated in the gel network? Furthermore, it is known that at pH values > 10 the dissolution of silica increases rapidly resulting in high silicate concentrations in the solution [68]. The silicates can deprotonate to produce a H^+^ and thus affect the pH but the effect of silicates on the aggregation of silica nanoparticles is not known to the authors.

The behaviour of silica sols at high pH levels is especially interesting from a grouting application point of view. Often when silica sols are used for grouting, this involves a mixed use of cement grout and silica grout. The resultant silica gel will thus be in contact with cement and the environment surrounding it. Given that cement contains alkaline hydroxides and calcium hydroxide in large amounts, these will leach out into the water leading to pH levels ranging from 12.0 to 13.5 [69, 70]. This might prove to be a problem for silica gels with potassium (structure breaker) as an accelerator since the gel can dissolve at this pH. The amount of covalent siloxane bonds between the silica nanoparticles will play a critical role whether potassium can be used as an accelerator in an environment of high pH i.e., above 11. Perhaps this explains the phenomenon mentioned in the introduction where the gel was observed to exit the boreholes in the form of a “mush”. The mix of silica gels and cement might also result in the formation of calcium silicate hydrate, due to the presence of Calcium, which forms a gel whose structure is dependent on the Ca/Si ratio [71].

It can be concluded that pH is an important factor when discussing the gelling of silica particles. It governs the surface charge of the particles and seems to affect the behaviour of different accelerators. It also affects what governs the particle stability; since at high pH DLVO theory is valid and at low pH non-DLVO phenomenon are present.

### Temperature effect on gelling of silica nanoparticles

Burton et al. [72] have shown that temperature has an effect on gel strength. They conducted shear stress tests on gels, which have shown that increase in temperature lead to stronger gels. There are two theories by which the temperature effects on gelling can be rationalized. The first theory is that the rate of siloxane bond formation between particles, already in the gel network, increases with increase in temperature [55]. The second theory is that the strength of the gel is dependent on the ratio of particles making up the gel. As has shown by Johnsson et al. [73] all of the particles and particle aggregates are not incorporated in the gel network at the gel point. The growth in strength could thereby be a result of further incorporation of particles and aggregates into the gel network. Increase in temperature should lead to more particles being incorporated into the gel network since diffusion speed of particles increases with temperature. It should also be mentioned that this increase in diffusion of particles with temperature leads to increased number of collision between particles which decrease the gel time, as reported by Huang et al. [58].

### Viscosity and structure development during gelling

**Fig. 6:**
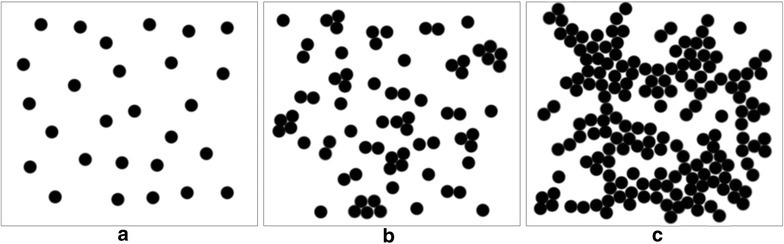
Shows the development of aggregates as the viscosity increase, where **a** represents silica sol before the introduction of accelerator where only single particles are present, **b** represents the formation of small aggregates shortly after introduction of accelerator, **c** represents the formation of a gel network as the PoG is reached. The viscosity can be said to be (**a**) < (**b**) < (**c**)

An increase in viscosity due to gel formation is well established [8, 58, 74]. The increase is due to the formation of aggregates as the particles are rendered unstable by the accelerator or changes in pH, see Fig. 6. As these aggregates form and grow the mobility of the individual particles and aggregates decrease leading to the increase in the viscosity. The formation of such aggregates has been observed [38].

The speed of the aggregate formation will govern the speed with which the viscosity increases. This is affected by the concentration and type of accelerator and ultimately whether the aggregation proceeds according to RLCA or DLCA mechanisms [53, 55, 58]. One hypothesis is that this is coupled to the strength development in the gel since a slower aggregation in accordance with RLCA mechanism could lead to closer packing of the particles, see Fig. 5b. However this is in conflict with the observation that silica sols show no volume change upon gelling [74–76].

### Methods for determining the PoG

The PoG is often used as a way to measure the effect that different parameters, such as accelerator type, particle size, and particle concentration, has on the formation of gel. The measurement of the PoG is therefore of importance if results are to be compared. In this part we review and discuss the methods used for PoG determination.

For the determination of the PoG two methods have been reported in the literature. The visual method is simple and easy to use [38, 58, 61, 73]. It needs no instrumentation since all that is required by the researcher/engineer is to determine when the silica sol no longer flows, as the vessel containing it is tipped to the side or upside down. The time it takes for the silica sol to reach the non-flowing state from the introduction of accelerator is taken as the gel time. A modified version of this method exists where a needle is inserted into the gel and the point at which the needle does not move upon tipping the gel is taken as the gel time [77]. Although this method is very easy to use it is not considered to be very scientific.

For a more scientific determination of the PoG rheological measurements using the Winters–Chambon criterion is used [53, 78, 79]. The method requires the measurement of the storage modulus (G′) and the loss modulus (G″) at a certain frequency (w) by oscillating viscosity measurements. The oscillating measurement setup is preferentially used instead of the more traditional cup and bob method since this setup does not break down the inter-particle silanol bindings of the gel. The Winters–Chambon criterion states that at the PoG the following power law is valid:

4 $$G^{\prime}(w)\sim G^{\prime\prime}(w)\sim w^{n}$$

where G′(w) is the storage modulus at frequency w, G″(w) is the loss modulus at frequency w, and n is the critical exponent. Using the Winters–Chambon criterion the PoG can be determined by establishing the point of intersection for the G′ and the G″ at the frequency w.

The choice of method can thus be a choice between simplicity, with the visual methods easy to use approach, or thoroughness, with the rheological measurements supported by the Winters–Chambon theory. Results have been produced Ågren and Rosenholm [80] where they compare these methods in a study of the phase behaviour and structure changes in tetraethylorthosilicate. They found that the difference in PoG for the two methods is negligible. This would suggest that even though the visual method is not considered very scientific the results produced by the method are close to the results that would be produced by the more scientific rheological method. Given the visual method’s ease of use it is understandable that many scientific groups choose this method when determining the PoG [38, 53, 58, 77].

## Mechanical properties of gelled silica sol

The mechanical property of the final gel is of critical importance for the materials application in grouting. Therefore in this section the gel strength, hydraulic conductivity, and durability of the gels will be discussed. These properties are connected to the previously discussed chemical properties of the silica sols and an attempt to bridge the gap between the two fields is made.

### Gel strength

When discussing the strength of silica gels it is important to define what is meant with gel strength since several different types of strength exist dependent on how the measurement of said strength has been conducted. Two examples of gel strength are shear strength and compressive strength [8, 72, 81]. The shear strength can be measured by fall-cone analysis and measures the resistance towards penetration of a pointy cone as it is dropped from a certain height into the silica. Compressive strength is the resistance towards shrinkage in volume of the gel as it is exposed to an external pressure. Axelsson [81], has shown that the compressive strength as well as the shear strength continues to develop for at least up to 6 months. From a chemical point of view the gel strength is dependent on the strength of the chemical bonds or interactions between the nanoparticles making up the gel. These bonds or interactions are at the heart of both the shear strength and the compressive strength since they govern the ease with which the gel deforms. When gel strength is mentioned further on it thus refers to the strength of these bonds or interactions between the nanoparticles, if not stated otherwise. A theoretical strength maximum should exist at which either: (1) no further siloxane bond formation is possible due to the exhaustion of bond sites between the particles or (2) all free particles or aggregates have been incorporated into the gel network. At this time no such maximum has been observed in the literature.

The gel strength is dependent on the structure of the particle network making up the gel. This can be coupled to the particle concentration of the gel [8]. A gel with a larger particle concentration contains closely packed particles that have plenty of contact areas between the particles. These contact areas are of critical importance since it is here that the chemical siloxane bonds between the particles are formed. A high amount of contact areas therefore lead to a stronger gel. These statements are supported in recent study [65], which showed that the structure of gel is highly dependent on the number of particles.

To some extent the gel strength should be governed by the particle size and particle distribution of the silica sol. The amount of surface area available for contact with other particles increase with decreased particle size and this has an effect on the ability to form stable siloxane bonds between the particles. The same is true for the polydispersity of the particles since a more polydispersed particle distribution enables particles to better fill out the available space and thus increase the contact area between particles. While none of this is as yet supported by literature it has been shown that polydispersed particles form more stable aggregates when compared to the aggregates of monodisperse particles [73]. It would be logical to assume that aggregate stability and strength are connected since they both depend on the particle interactions.

The effect of temperature on gel strength has been discussed previously in this review. As a summary on the effect of temperature on gel strength it can be said that increase in temperature leads to stronger gel, since increase in temperature lead to increased formation of siloxane bonds between particles.

The choice of accelerator has an effect on the gel strength as pointed out in “Effects of ion type on the gelling of silica” section. In two studies [54] and [32] it has been shown that the greatest gel strength at pH intervals of 8–10 is achieved using the structure breaker ions. One explanation for this might be the closer adsorption of the ion to the surface of the silica leading to shorter distances between aggregated particles. This in turn will ease bond formation between particles resulting in a stronger gel. However, studies of the aggregation of silica sols seem to point in the other direction. While the most stable aggregates were indeed produced by the structure breaker ions, the rate of strength development was shown to be greater for sodium, a structure maker ion [38, 61, 65]. These studies would seem to be in agreement with the observations on viscosity development, where the highest viscosity was observed for structure maker ions [37]. The antagonistic behaviour of accelerators, previously discussed, should also be mentioned since at pH levels above 11 the gels formed with structure breaker ions might break apart and from a grouting perspective lead to complete failure of the grout. It is therefore unclear what the effect of the accelerator of gel strength is since results seem to diverge. We can conclude that further studies into the strength developments dependency on the choice of accelerator are needed.

### Hydraulic conductivity in silica nanoparticle gels

**Fig. 7:**
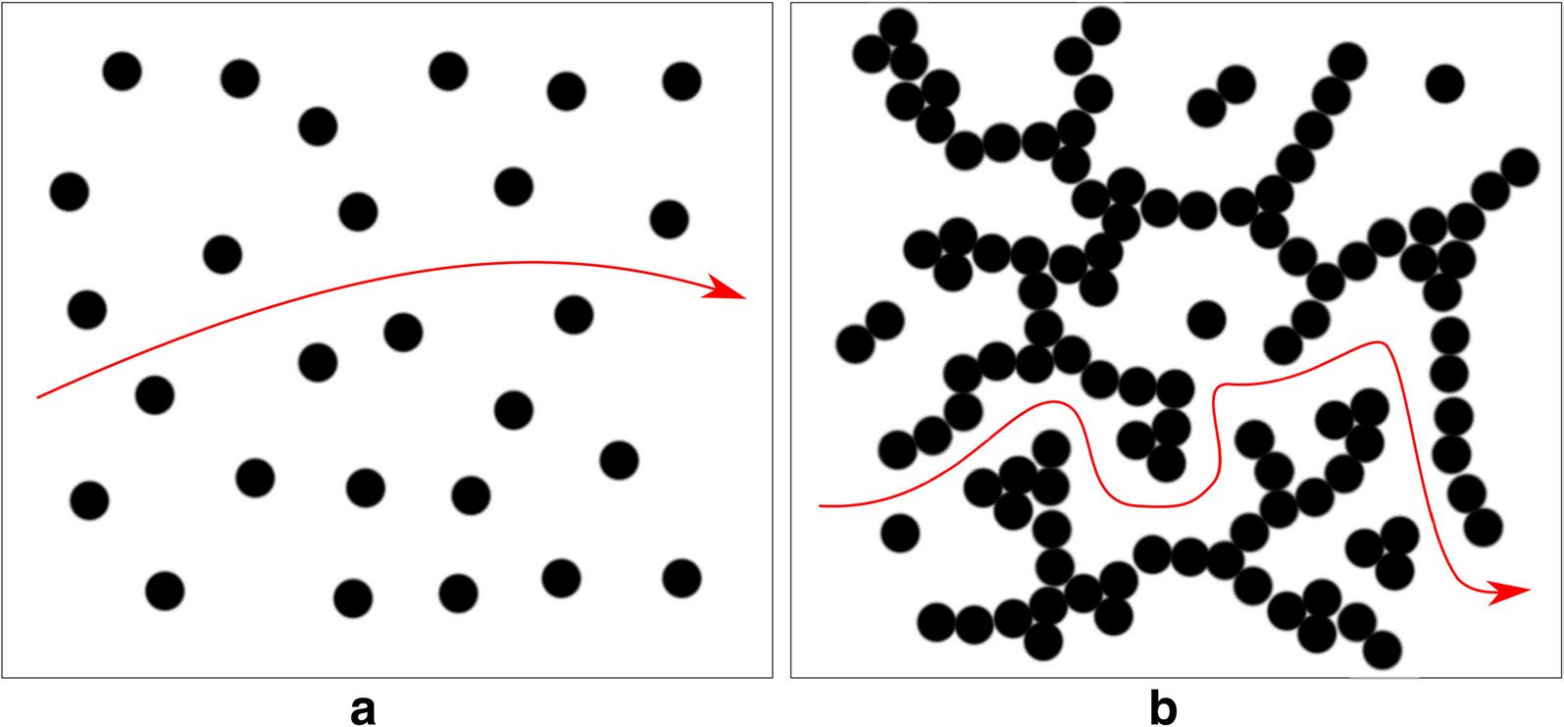
The red lines in **a** and **b** clearly shows the difference in distance travelled by water molecules as they pass through the gel. **a** Is much shorter and represents silica sol before the addition of accelerator and the longer, **b** represents gelled silica

One of the properties that enable gelled silica sol to function as a grouting material is its low hydraulic conductivity. For silica gels the value may vary dependent on the concentration of silica particles in the original sol. Silica sols with a concentration above 7.4wt% was shown [8] to meet the required value for barrier materials set to a maximum of 10^−7^ cm/s. Tests conducted by Butrón et al. [72] have shown that the hydraulic conductivity decrease with time and can be as low as 10^−8^–10^−9^ cm/s. The low hydraulic conductivity can be explained by analyzing the distance a water molecule travels through the gel, see Fig. 7. The gel is made up of a amorphous network of silica nanoparticles with water dispersed in pores in between the particles. These water pores twist and turn at random and can be described as a maze that the water molecule must travel in order to pass through the gel. The distance travelled by each water molecule is thus much greater than the thickness of the gel. The decrease in hydraulic conductivity with time can be attributed to the continued buildup of the particle network. Additional twists and turns are thus added to the maze over time increasing the travel distance of individual water molecules. This also explains the effects observed as a result of particle concentration. A silica sol with higher particle concentration leads to a maze with more twists and turns than a silica sol with lower particle concentration. Also since the silica network will carry a negative charge the presence of ions in the water might slow down the flow through the gel. The ions will be attracted to the silica surface and a flow gradient will form where ions close to the surface travel slower, not unlike that observed in for example ion cromatography.

### Durability and longevity of silica nanoparticle gels

Little can be said about the durability and longevity of silica gels. [75, 76] reported very low leaching of silica, maximum 1–2%, from gelled colloidal silica with a particle size of 10 nm but the test parameters were not reported. It was also reported that, as mentioned previously, colloidal silica gels continues to grow in strength throughout the test period of 90 days and that no strength maximum was observed. The only deviation from this is at strong alkaline conditions where the gel strength decreased perhaps due to the antagonistic effect described previously. From this the conclusion can be drawn that the durability and longevity of silica gels are coupled to the surrounding environment in which they are deployed. The importance of choosing the right accelerator depends on the surrounding environment. For example at strong alkali (pH > 11) structure maker ions might prove to be more effective in producing a durable gel due to the previously described antagonistic effect.

This brings us to question what effect the ion composition surrounding the gel might have on the durability? For example imagine a scenario where sodium is used as an accelerator and the pH of the surrounding environment is 13. Under pure conditions where the surrounding water is free of other ions this should not prove a problem for the gel. But for grouting application the groundwater is never free of ions. So what happens if the groundwater contains potassium ions? Will there be an ion exchange in the gel in which the sodium ions are exchanged with potassium ions? If this is the case, the gel might be rendered unstable and start to dissolve. We have not been able to find any literature that answers these questions and therefore these remain as open questions.

The choice of accelerator can also impact the gel time and often silica sols are supplied with a suggested mixing ratio and therefore a suggested gel time. Shen et al. have reported dissolution and erosion behaviour of gels made from three different sols; namely Cembinder, Eka EXP36, and MEYCO MP320 [77]. These sols have different particle size distributions and were tested with gel times suggested by the supplier. It was shown that the MEYCO gel had the longest gel time and also suffered most from dissolution and erosion compared to the other two gels which had shorter gel times. We think that since gel time also represents strength build-up in the gels, this might partly explain these results. Since a gel with shorter gel time will have more time to establish and build-up strength through the continued incorporation of particles into the gel network and formation of siloxane bonds between particles; it is not surprising that these gels show the least amount of erosion.

## Conclusions

Silica gels created from silica nanoparticles is a complex interdisciplinary subject. It stretches from the scientific fields of surface chemistry and colloid chemistry to the engineering field of material technology and, when used for grouting, geo-technology. The grouting area of application means that the gels are exposed to an environment where pH, water composition, and water pressure may vary. This puts a high demand on gel performance and increases the importance of understanding the impact of these factors as well as the impact of choice of silica sol and accelerator.

In this review we have tried to bring together the knowledge available so far in the two fields of colloidal science and tunnel grouting. We have methodically worked our way through the different factors that may affect the silica gels, from the availability of different silica sols to the impact of environmental factors. We have found that these factors play an integrate part for the effectiveness of the silica gels as a grouting material. Also the behaviour of the gels can be predicted by understanding the chemistry of the key building block; the silica nanoparticles. This understanding will be critical for the future designing and refinement of silica sols for grouting purposes.
